# Correlation between LSM1 Expression and Clinical Outcomes in Glioblastoma and Functional Mechanisms

**DOI:** 10.1155/2023/1543620

**Published:** 2023-11-02

**Authors:** Changcheng Cai, Xingyu Chen, Jimin He, Chengwei Xiang, Yinggang Liu, Ke Wu, Ke Luo

**Affiliations:** ^1^Department of Neurosurgery, Suining Central Hospital, Suining, 629000 Sichuan, China; ^2^Department of Neurosurgery, Xichang People's Hospital, Xichang, 615000 Sichuan, China

## Abstract

**Background:**

Glioblastoma (GBM) is an aggressive form of brain tumor characterized by limited treatment options and a bleak prognosis. Although the role of Like-Sm 1 (LSM1), a component of the mRNA splicing machinery, has been studied in various cancers, its significance in GBM remains unclear. The purpose of this research was to investigate the expression of LSM1 and its role in driving GBM progression.

**Methods:**

We analyzed gene expression data obtained from TCGA and GTEx databases to compare the levels of LSM1 expression between GBM and normal brain tissues. To assess the impact of LSM1, we conducted experiments using U87 GBM cells, wherein we manipulated LSM1 expression through overexpression and knockdown techniques. These experiments allowed us to evaluate cellular behaviors such as proliferation and invasion. Additionally, we explored the correlation between LSM1 expression and immune cell infiltration in GBM.

**Results:**

Our analysis of TCGA and GTEx datasets revealed a significant upregulation of LSM1 expression in GBM compared to normal brain tissues. In our in vitro experiments using U87 cells, we observed that LSM1 overexpression promoted cell proliferation and invasion, while LSM1 knockdown exerted the opposite effects. Moreover, we discovered correlations between LSM1 expression and immune cell infiltration in GBM, specifically involving TFH cells, CD56bright cells, macrophages, and Th2 cells.

**Conclusions:**

The findings of this study demonstrate the upregulation of LSM1 in GBM and its contribution to tumor progression by enhancing cell proliferation, invasion, and influencing immune cell infiltration. Our research sheds light on the potential oncogenic role of LSM1 in GBM and suggests its viability as a therapeutic target for this aggressive brain tumor.

## 1. Introduction

Glioma, the most prevalent primary malignant brain tumor, includes glioblastoma (GBM) as its highly aggressive subtype [1]. GBM is characterized by rapid proliferation, invasive growth, and resistance to standard therapies, leading to a grim prognosis for patients [2]. Consequently, it is crucial to comprehend the molecular mechanisms driving GBM progression in order to develop innovative therapeutic strategies [3].

Among the intriguing molecular factors, LSM1 (Like-Sm 1), a member of the Sm-like (LSm) protein family, has gained attention. LSm proteins, which are evolutionarily conserved, play roles in diverse aspects of RNA metabolism, including RNA splicing, degradation, and translational regulation [4]. LSM1, specifically, is involved in the processing and degradation of noncoding RNAs such as microRNAs and long noncoding RNAs, as well as mRNA decay and surveillance [5, 6].

In addition to its established involvement in RNA metabolism, emerging evidence suggests that LSM1 may possess functions relevant to tumorigenesis. Several studies have documented dysregulated expression of LSM1 in various cancer types, including lung cancer [7], prostate cancer [8], and breast cancer [9], indicating its potential as an oncogenic driver promoting tumor cell proliferation, invasion, and metastasis in multiple malignancies [10]. Despite these findings in other cancers, the precise role of LSM1 in glioblastoma remains largely unexplored. Given the aggressive nature of GBM and the urgent need for novel therapeutic targets, it is essential to investigate the functional significance of LSM1 in GBM progression.

Therefore, the objective of this study was to examine LSM1 expression patterns and its role in GBM. We performed a comprehensive analysis of LSM1 expression levels in a cohort of GBM patients and investigated its associations with clinicopathological features such as patient age, gender, IDH status, and 1p/19q codeletion. Furthermore, we aimed to determine the potential impact of LSM1 on GBM cell proliferation, invasion, and migration, providing valuable insights into its oncogenic potential and its suitability as a therapeutic target in GBM.

## 2. Methods

### 2.1. Online Dataset Search and Information Retrieval

To acquire gene expression data and clinical information, we accessed publicly available online datasets. The Cancer Genome Atlas (TCGA) and Genotype-Tissue Expression (GTEx) databases provided the necessary gene expression data for LSM1 in glioblastoma (GBM) and normal brain tissues, respectively. Detailed patient characteristics, including age, gender, IDH status, and 1p/19q codeletion, were obtained from TCGA dataset.

### 2.2. U87 Cell Culture and Manipulation of LSM1 Expression

The U87 GBM cell line was procured from ATCC (American Type Culture Collection) and cultured in suitable growth media supplemented with fetal bovine serum and antibiotics. For LSM1 overexpression, U87 cells were transfected with an LSM1 expression vector using a transfection reagent as per the manufacturer's instructions. Conversely, for LSM1 knockdown, U87 cells were transfected with specific small interfering RNA (siRNA) targeting LSM1 or non-targeting scrambled siRNA as a control [11]. The efficiency of overexpression and knockdown was assessed through Western blotting.

### 2.3. Western Blot

The proteins extracted from whole cell lysates using SDS-lysis buffer were separated on SDS-PAGE gels and subsequently transferred onto PVDF membranes. These membranes were then blocked with Tween-Tris-buffered saline (TTBS) for a duration of 2 hours at room temperature, followed by incubation with primary antibodies. Specifically, the primary antibodies used were anti-LSM1 (ab229316, Abcam, diluted 1 : 10000) and anti-beta-actin (sc-47778, Santa Cruz Biotechnology, diluted 1 : 10000). Following this, the membranes were incubated with secondary antibodies for 2 hours at room temperature. The visualization of immunoblots was achieved using an enhanced chemiluminescence (ECL) kit (Beyotime, Shanghai, China), and subsequent analysis involved the scanning of blot bands.

### 2.4. CCK-8 Assays

To evaluate cell proliferation, we performed cell counting assays using the cell counting Kit-8 (CCK-8) method. U87 cells with LSM1 overexpression or knockdown, along with corresponding control cells, were seeded in 96-well plates at defined densities (3000 cells/well). After the desired incubation period (24, 48, 72, and 96 hours), the CCK-8 reagent was added to each well, and the absorbance was measured using a microplate reader. The absorbance values were then used to assess cell proliferation rates [12].

### 2.5. Matrigel-Transwell Experiments

Matrigel-transwell experiments were conducted to assess cell invasion capabilities. U87 cells with LSM1 overexpression or knockdown, as well as control cells, were seeded onto the upper chamber (200000 cells/chamber) of transwell inserts precoated with matrigel (80 *μ*l, BD, Franklin Lakes, NJ, USA). In the lower compartment, 600 *μ*l of 10% FBS medium was added as a chemoattractant. Following the incubation period, cells that invaded through the matrigel and migrated to the lower surface of the membrane were fixed, stained, and counted under a microscope [13].

### 2.6. Statistical Analyses

The statistical analyses were conducted using the SPSS.22.0 software package. To determine the significance of differences in LSM1 expression between GBM and normal brain tissues, Student's t-test or non-parametric tests were employed, depending on the data distribution. Survival analyses were performed using the Kaplan-Meier method, and differences between survival curves were assessed using log-rank tests. The association between LSM1 expression and clinicopathological characteristics was evaluated using chi-square tests or Fisher's exact tests. In the case of in vitro experiments, statistical significance was determined using t-tests or one-way analysis of variance (ANOVA), followed by post hoc tests if necessary. A *p* value below 0.05 was considered statistically significant [14].

### 2.7. Ethics

This study was conducted in compliance with ethical guidelines and regulations. The utilization of publicly available datasets adhered to the data access policies and informed consent procedures established by TCGA and GTEx projects. The ethics committee of our center confirmed that this study does not need animal or human research approval.

## 3. Results

### 3.1. LSM1 Expression in TCGA and GTEx Dataset

**Figure 1:**
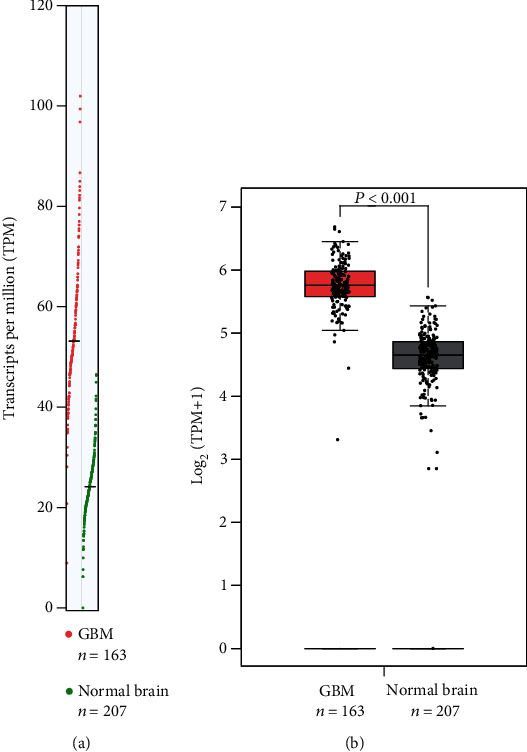
Comparative analysis of LSM1 expression in glioblastoma (GBM) and normal brain tissues. Comparison of LSM1 expression levels between GBM (*N* = 163) and normal brain tissues (*N* = 207) using TCGA and GTEx datasets. (a) A box plot representation of LSM1 expression levels in GBM and normal brain tissues is presented, measured in transcripts per million (TPM). The box plot visualizes the distribution of expression levels, with the median indicated by a horizontal line within the box. Outliers are represented as individual data points. (b) A bar graph displays the statistical significance of the differential LSM1 expression between GBM and normal brain tissues. The figure emphasizes the significantly higher expression of LSM1 in GBM compared to normal brain tissues.

We firstly conducted the comparative analysis of LSM1 expression levels in glioblastoma (GBM) and normal brain tissues (Figure 1). The data were derived from TCGA (The Cancer Genome Atlas) and GTEx (Genotype-Tissue Expression) datasets, encompassing 163 GBM samples and 207 normal brain tissue samples. The LSM1 expression levels were quantified as transcripts per million (TPM). Both Figures 1(a) and 1(b) clearly demonstrate a significant increase in LSM1 expression in GBM compared to normal brain tissues. The TPM values for LSM1 in GBM samples exhibited a substantial elevation compared to those in normal brain tissues. This finding indicates a pronounced upregulation of LSM1 in GBM, suggesting its potential role in the pathogenesis of this aggressive brain tumor.

**Figure 2:**
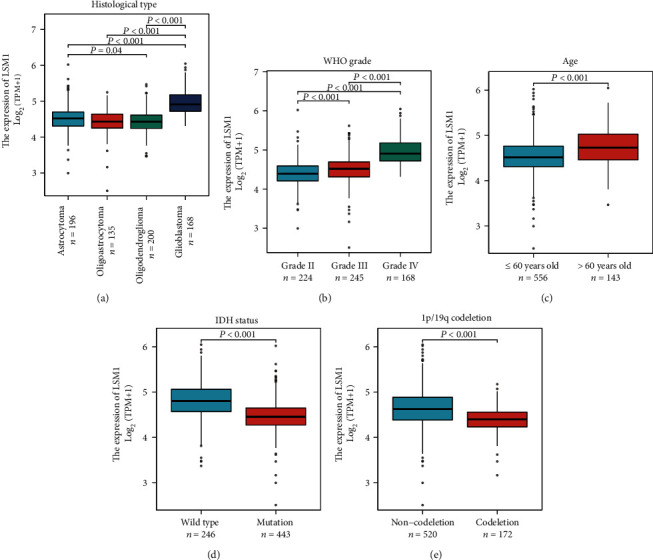
Comparison of LSM1 expression levels in different glioma subtypes. This figure showcases the analysis of LSM1 expression levels in glioma subtypes based on histological type (a), WHO grade (b), age (c), IDH status (d), and 1p/19q codeletion (e). Bar graphs depict the relative expression of LSM1 in each glioma subgroup. The findings demonstrate a marked increase in LSM1 expression in glioblastoma (a), higher WHO grades (b), older age (c), wild-type IDH status (d), and gliomas without 1p/19q codeletion (e). These results suggest a potential association between LSM1 upregulation and more aggressive glioma characteristics (all *p* < 0.05), highlighting its promise as a prognostic biomarker and a potential therapeutic target.

We next investigated the levels of LSM1 expression in different glioma subtypes based on histological type, WHO grade, age, IDH status, and 1p/19q codeletion, which demonstrate the significant elevation of LSM1 expression in specific glioma subgroups (Figure 2). In Figure 2(a), LSM1 expression was found to be significantly higher in glioblastoma, the most aggressive histological type, compared to other glioma types. Similarly, in Figure 2(b), LSM1 levels showed a significant increase with higher WHO grades, with grade IV gliomas exhibiting the highest LSM1 expression.  Figure 2(c) revealed that elder age was associated with elevated LSM1 expression in gliomas. In Figure 2(d), wild-type IDH status was linked to significantly higher LSM1 expression compared to IDH-mutant gliomas. Finally, Figure 2(e) demonstrated that gliomas without 1p/19q codeletion displayed significantly higher LSM1 expression levels compared to those with codeletion. These findings suggest that LSM1 upregulation may be a characteristic feature of more aggressive and clinically unfavorable gliomas, highlighting its potential role as a biomarker for disease prognosis and as a target for therapeutic interventions.

### 3.2. Patients' Characteristics

Table 1 presents the patient characteristics categorized by low and high expression of LSM1. The study involved 349 cases with low LSM1 expression and 350 cases with high LSM1 expression. The age distribution was statistically significant on that younger patients were more prevalent to show low LSM1 level (*p* < 0.001). There was no significant difference in gender between the two groups. Nonetheless, significant differences were observed in IDH status and 1p/19q codeletion (*p* < 0.001 for both). Consistent with Figure 1, the low LSM1 expression group had fewer cases with IDH mutation or 1p/19q codeletion, while the high LSM1 expression group had more cases.

### 3.3. Survival Analysis Based on LSM1

**Figure 3:**
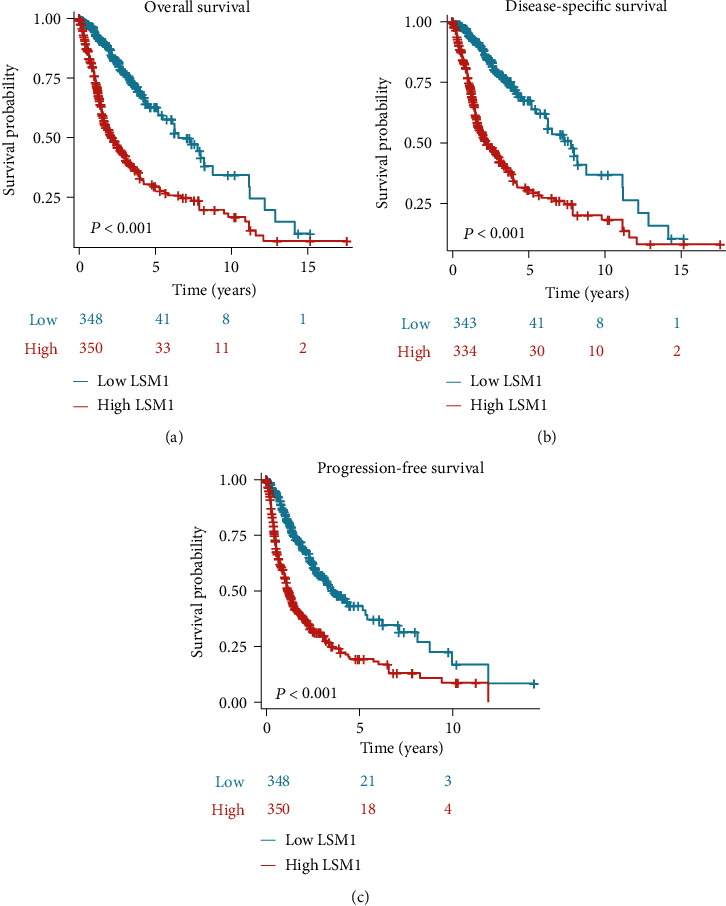
Impact of LSM1 expression on survival outcomes in glioma patients. The analysis of overall survival (a), disease-specific survival (b), and progression-free survival (c) based on LSM1 expression levels in glioma patients was conducted. Kaplan-Meier survival curves are depicted for patients with either high or low LSM1 expression. The consistent results demonstrate that patients with higher LSM1 expression exhibit significantly worse clinical outcomes in terms of overall survival (a), disease-specific survival (b), and progression-free survival (c).

To better evaluate the clinical impact of LSM1 expression, we next tested its effect on overall survival (Figure 3(a)), disease-specific survival (Figure 3(b)), and progression-free survival (Figure 3(c)) in glioma patients. The results consistently demonstrate that patients with higher LSM1 expression levels exhibit significantly worse clinical outcomes. In Figure 3(a), the survival analysis reveals a clear association between higher LSM1 expression and reduced overall survival, indicating a poorer prognosis for patients with elevated LSM1 levels. This trend is further supported by Figure 3(b), which demonstrates that higher LSM1 expression is associated with a significant decrease in disease-specific survival, emphasizing the impact of LSM1 on glioma-specific mortality. Additionally, Figure 3(c) illustrates that patients with higher LSM1 expression have shorter progression-free survival, indicating a higher likelihood of disease progression in this subgroup. Collectively, these findings indicate the prognostic significance of LSM1 in glioma, with increased LSM1 expression serving as an indicator of poorer clinical outcomes.

Additionally, univariate and multivariate analyses were performed to assess the prognostic significance of other clinical pathological characteristics (Table 2). Age was found to be a significant prognostic factor. Patients older than 60 years had a higher hazard ratio (HR) of 4.696 (95% CI: 3.620-6.093, *p* < 0.001) compared to those aged 60 or younger. This association remained significant in the multivariate analysis, with an HR of 2.054 (95% CI: 1.541-2.737, p <0.001). Gender, on the other hand, did not show a significant association with survival. Consistent with previous findings, IDH mutation status was strongly associated with patient prognosis. Patients with IDH mutation had a lower HR of 0.116 (95% CI: 0.089-0.151, *p* < 0.001) in the univariate analysis, indicating a better prognosis. This association remained significant in the multivariate analysis, with an HR of 0.183 (95% CI: 0.132-0.253, *p* < 0.001). Similarly, the presence of 1p/19q codeletion was associated with a better prognosis, with a lower HR of 0.225 (95% CI: 0.147-0.346, *p* < 0.001) in the univariate analysis. However, in the multivariate analysis, the association became marginally significant (*p* = 0.065). As described before, high expression of LSM1 was significantly associated with a poorer prognosis. In the univariate analysis, patients with high LSM1 expression had a higher HR of 2.878 (95% CI: 2.217-3.735, *p* < 0.001). This association remained significant in the multivariate analysis, with an HR of 1.473 (95% CI: 1.114-1.949, *p* = 0.007), highlighting the clinical significance of LSM1 in predicting GBM survival.

### 3.4. LSM1 Enhances Glioblastoma Cell Growth and Invasion

**Figure 4:**
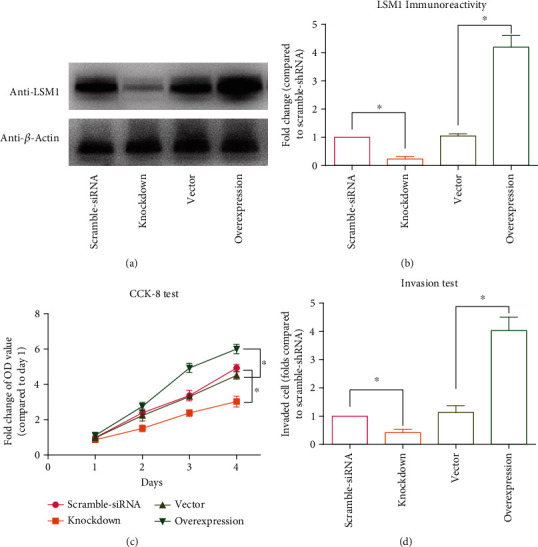
Effects of LSM1 modulation on U87 GBM cell behavior. Cellular consequences of LSM1 modulation in U87 GBM cells were evaluated. (a) Western blot analysis confirms the efficiency of LSM1 knockdown and overexpression, using scrambled-siRNA and vector controls, respectively. (b) Semiquantification of the western blot results demonstrates successful LSM1 knockdown and overexpression. (c) The impact on cell proliferation is evaluated using the CCK-8 assay, revealing that LSM1 overexpression promotes cell proliferation, while knockdown of LSM1 inhibits cell growth in U87 GBM cells. (d) Cell invasion capacity is assessed, indicating that LSM1 overexpression enhances GBM cell invasion, while LSM1 knockdown significantly inhibits this process. These findings elucidate the functional implications of LSM1 in GBM, where overexpression promotes cell proliferation and invasion, while knockdown exerts suppressive effects. LSM1 emerges as a potential therapeutic target for GBM. ^∗^ indicates *p* < 0.05 by the Student's *t*-test between the two groups.

The effects of LSM1 overexpression and knockdown on U87 glioblastoma (GBM) cells were further examined (Figure 4). The efficiency of LSM1 knockdown and overexpression was assessed using western blotting, with scrambled-siRNA and vector serving as controls, respectively (Figure 4(a)). The semiquantitative results in Figure 4(b) confirm the successful modulation of LSM1 expression, as indicated by the significant reduction or increase in LSM1 protein levels compared to the control groups.

Cell proliferation was evaluated using the CCK-8 assay. The data demonstrate that LSM1 overexpression promotes cell proliferation in U87 GBM cells, while LSM1 knockdown exerts an inhibitory effect on cell growth (Figure 4(c)). Furthermore, we investigate the influence of LSM1 on cell invasion. The results indicate that LSM1 overexpression enhances the invasive potential of GBM cells, whereas LSM1 knockdown significantly inhibits cell invasion (Figure 4(d)). These observations suggest that LSM1 plays a critical role in promoting the viability and invasive capabilities of GBM cells, thereby contributing to the aggressive behavior of this malignancy. The cellular data provide compelling evidence for the functional significance of LSM1 in GBM, suggesting that LSM1 may represent a promising therapeutic target in GBM and highlighting its potential implications for GBM treatment strategies.

### 3.5. LSM1 Modulates Immune Cell Infiltration in Tumor Microenvironments

**Figure 5:**
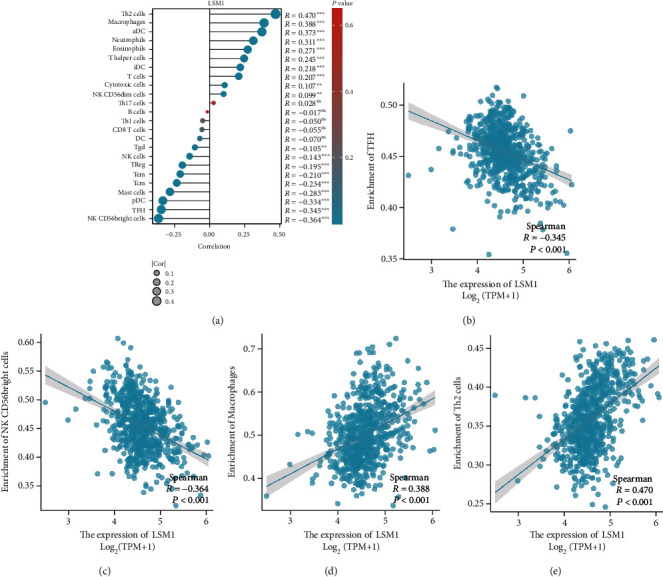
Correlation between LSM1 expression and immune cell infiltration in GBM using TCGA dataset. (a) Analysis of LSM1 expression reveals correlations with immune cell infiltration in GBM. Representative correlations are shown: (b) negative correlation with TFH cell infiltration; (c) negative correlation with CD56bright cell infiltration; (d) positive correlation with macrophage infiltration; and (e) positive correlation with Th2 cell enrichment. These findings indicate that LSM1 expression in GBM is linked to distinct patterns of immune cell infiltration, shed light on the potential immunoregulatory role of LSM1 in the GBM tumor microenvironment, and provide insights into its impact on immune cell interactions.

The association between LSM1 expression and immune cell infiltration in GBM was examined using data from TCGA dataset. The analysis explores the correlations between LSM1 expression and multiple immune cell infiltration in GBM (Figure 5(a)), and Figures 5(b)–5(e) highlight specific correlations with representative immune cell populations. For example, Figure 5(b) reveals a negative correlation between LSM1 expression and TFH (T follicular helper) cell infiltration, suggesting that higher LSM1 expression may be associated with reduced TFH cell infiltration in GBM. Similarly, Figure 5(c) demonstrates a negative correlation between LSM1 expression and CD56bright cell infiltration, indicating that increased LSM1 expression may be linked to decreased infiltration of CD56bright cells in GBM.

Conversely, we found a positive correlation between LSM1 expression and macrophage infiltration, suggesting that higher LSM1 expression may contribute to increased macrophage infiltration in GBM (Figure 5(d)). Furthermore, a positive correlation between LSM1 expression and Th2 cell enrichment (Figure 5(e)) was observed, indicating that elevated LSM1 expression may be associated with enhanced Th2 cell infiltration in GBM.

Overall, the findings suggest that LSM1 expression in GBM is associated with specific immune cell infiltration patterns, which provides insights into the potential immunoregulatory role of LSM1 in GBM, highlighting its involvement in modulating the tumor microenvironment and immune cell responses. Further investigation is necessary to elucidate the underlying mechanisms driving these correlations and to explore the clinical implications of LSM1-mediated immune cell interactions in GBM.

## 4. Discussions

Glioblastoma is an aggressive and lethal brain tumor known for its rapid growth, infiltrative behavior, and resistance to treatment. To advance our understanding of GBM progression, this study focused on investigating the oncogenic role of LSM1. The results of our study revealed significantly higher LSM1 expression levels in GBM tissues compared to normal brain tissues. This finding aligns with previous research in lung cancer, breast cancer, and pancreatic cancer, which also reported dysregulated LSM1 expression [9, 10, 15, 16]. LSM1 is involved in various cellular processes, including mRNA splicing, degradation, and translation. Its interaction with multiple proteins in mRNA decay machinery suggests its potential role in posttranscriptional regulation [17–19]. The upregulation of LSM1 in GBM may contribute to dysregulated RNA metabolism and altered gene expression patterns associated with tumor progression.

Additionally, our study demonstrated that high LSM1 expression in GBM was associated with unfavorable clinicopathological characteristics. These characteristics included older age, wild-type IDH status, and noncodel status. Similar associations have been observed in other cancers, further supporting LSM1 as a prognostic marker. For example, elevated LSM1 expression in lung cancer is correlated with advanced tumor stage and reduced patient survival [20]. In contrast, LSM1 seems to play anti-tumor effects in prostate cancer [8]. These findings underscore the potential of LSM1 as a prognostic biomarker in GBM, facilitating patient stratification and personalized treatment decisions.

Our survival analysis demonstrated that high LSM1 expression was significantly associated with worse overall survival, disease-specific survival, and progression-free survival in GBM patients. This suggests the potential of LSM1 as a prognostic indicator and a predictive marker for treatment response in GBM. Similar prognostic significance of LSM1 has been observed in other cancers, where elevated LSM1 expression is correlated with reduced overall survival and disease-free survival. These results highlight the potential of LSM1 as a promising therapeutic target and emphasize the need for further investigation into its functional implications in GBM progression.

Functional experiments conducted in U87 GBM cells are aimed at investigating the effects of LSM1 on cell proliferation and invasion. Accordingly, the results indicated that LSM1 overexpression promoted cell proliferation and invasion, while LSM1 silencing had the opposite effect. These findings are consistent with previous studies that have implicated LSM1 in cancer cell growth and invasion. For example, in breast cancer, LSM1 knockdown resulted in inhibited cell proliferation, migration, and invasion, highlighting its role in regulating tumor cell behavior [9]. Mechanistically, LSM1 has been shown to interact with various proteins involved in cell cycle regulation, apoptosis, and epithelial-mesenchymal transition (EMT), emphasizing its multifaceted involvement in cancer progression [21].

Furthermore, our study explored the relationship between LSM1 expression and immune cell infiltration in GBM. We observed significant correlations between LSM1 expression and specific immune cell populations, including TFH cells, CD56bright cells, macrophages, and Th2 cells. These findings suggest a potential immunomodulatory role of LSM1 in the tumor microenvironment. Immune cell infiltration plays a crucial role in tumor progression and treatment response [22]. Dysregulation of LSM1 may contribute to immune evasion mechanisms employed by GBM, influencing the tumor's interaction with the immune system and shaping the immunosuppressive microenvironment, which had also been reported in breast cancer [23]. Further investigations are required to unravel the underlying mechanisms driving these correlations and explore the therapeutic implications for immunotherapy strategies in GBM [24].

Despite the significant findings and implications of our study, it is important to acknowledge several limitations. Firstly, the retrospective nature of our analysis utilizing public databases may introduce selection bias and potential confounding factors. Additionally, the sample size of our experimental studies using GBM cell lines was relatively small, necessitating further validation in larger cohorts and in vivo models. Furthermore, although we investigated the association between LSM1 expression and immune cell infiltration in GBM, the functional consequences and underlying mechanisms remain to be elucidated. Future studies should incorporate mechanistic investigations to provide a comprehensive understanding of LSM1's role in modulating immune responses in the tumor microenvironment. Lastly, our study focused on LSM1 as a single biomarker, and it is important to consider the contribution of other molecular alterations and signaling pathways in GBM progression.

Despite these limitations, our study sheds light on the oncogenic role of LSM1 in GBM and lays the foundation for future research in this field. We systematically analyzed gene expression data from extensive databases, uncovering a notable upregulation of LSM1 in GBM tissues compared to normal brain samples, hinting at its potential as a promising biomarker for assessing disease prognosis. Further investigations revealed correlations between LSM1 expression and critical clinicopathological features such as patient age, WHO grade, IDH status, and 1p/19q codeletion, emphasizing its role as a prognostic tool. Functional experiments in U87 GBM cells underscored LSM1's impact on cell proliferation and invasion, solidifying its candidacy as a therapeutic target. Our exploration also delved into LSM1's involvement in immune cell infiltration in GBM, revealing intriguing correlations that suggest its role in shaping the tumor microenvironment and influencing immune responses [25]. However, the study acknowledges its limitations, including its retrospective nature and small experimental sample size, necessitating further mechanistic investigations to fully comprehend LSM1's multifaceted role in GBM tumorigenesis. Despite these challenges, this study represents a significant stride in unraveling GBM's complexities, offering a glimpse into LSM1's potential as a crucial player in disease progression and treatment possibilities.

## 5. Conclusions

Our study provides novel insights into the oncogenic role of LSM1 in promoting GBM progression. We observed elevated LSM1 expression in GBM tissues, which was associated with adverse clinicopathological characteristics and poor patient outcomes. Functional experiments confirmed LSM1's involvement in modulating cell proliferation, invasion, and immune infiltration in GBM.

## Figures and Tables

**Table 1:** Patients' characteristics.

Characteristics	Low expression of LSM1	High expression of LSM1	*p* value
Total cases (n)	349	350	
Age, *n* (%)			<0.001 ^∗∗∗^
≤60 years old	301 (43.1%)	255 (36.5%)	
>60 years old	48 (6.9%)	95 (13.6%)	
Gender, *n* (%)			0.062
Female	161 (23%)	137 (19.6%)	
Male	188 (26.9%)	213 (30.5%)	
IDH status, (%)			<0.001 ^∗∗∗^
Wild type	61 (8.9%)	185 (26.9%)	
Mutation	287 (41.7%)	156 (22.6%)	
1p/19q codeletion, *n* (%)			<0.001 ^∗∗∗^
Noncodel	218 (31.5%)	302 (43.6%)	
Codel	131 (18.9%)	41 (5.9%)	

^∗∗∗^ *P* < 0.001.

**Table 2:** Survival analysis of glioma patients.

Characteristics	Cases (*n*)	Univariate analysis		Multivariate analysis	
		Hazard ratio (95% CI)	*p* value	Hazard ratio (95% CI)	*p* value
Age (years old)	698		<0.001 ^∗∗∗^		
≤60	555	Reference		Reference	
>60	143	4.696 (3.620-6.093)	<0.001 ^∗∗∗^	2.054 (1.541-2.737)	<0.001 ^∗∗∗^
Gender	698		0.071		
Female	297	Reference			
Male	401	1.250 (0.979-1.595)	0.073		
IDH status	688		<0.001 ^∗∗∗^		
Wild type	246	Reference		Reference	
Mutation	442	0.116 (0.089-0.151)	<0.001 ^∗∗∗^	0.183 (0.132-0.253)	<0.001 ^∗∗∗^
1p/19q codeletion	691		<0.001 ^∗∗∗^		
Noncodel	520	Reference		Reference	
Codel	171	0.225 (0.147-0.346)	<0.001 ^∗∗∗^	0.634 (0.390-1.028)	0.065
LSM1	698		<0.001 ^∗∗∗^		
Low	348	Reference		Reference	
High	350	2.878 (2.217-3.735)	<0.001 ^∗∗∗^	1.473 (1.114-1.949)	0.007 ^∗∗^

^∗∗^ *P* < 0.01, ^∗∗∗^*P* < 0.001.

## Data Availability

Data will be available upon request.
